# Neuronal Growth and Behavioral Alterations in Mice Deficient for the Psychiatric Disease-Associated *Negr1* Gene

**DOI:** 10.3389/fnmol.2018.00030

**Published:** 2018-02-09

**Authors:** Katyayani Singh, Desirée Loreth, Bruno Pöttker, Kyra Hefti, Jürgen Innos, Kathrin Schwald, Heidi Hengstler, Lutz Menzel, Clemens J. Sommer, Konstantin Radyushkin, Oliver Kretz, Mari-Anne Philips, Carola A. Haas, Katrin Frauenknecht, Kersti Lilleväli, Bernd Heimrich, Eero Vasar, Michael K. E. Schäfer

**Affiliations:** ^1^Department of Physiology, Institute of Biomedicine and Translational Medicine, University of Tartu, Tartu, Estonia; ^2^Centre of Excellence in Genomics and Translational Medicine, University of Tartu, Tartu, Estonia; ^3^Department of Neuroanatomy, Institute of Anatomy and Cell Biology, Faculty of Medicine, University of Freiburg, Freiburg, Germany; ^4^Department of Anesthesiology, University Medical Center, Johannes Gutenberg-University Mainz, Mainz, Germany; ^5^Institute of Neuropathology, University Medical Center, Johannes Gutenberg-University of Mainz, Mainz, Germany; ^6^Focus Program Translational Neurosciences, Johannes Gutenberg-University of Mainz, Mainz, Germany; ^7^Mouse Behavioral Unit, Johannes Gutenberg-University of Mainz, Mainz, Germany; ^8^Experimental Epilepsy Research, Department of Neurosurgery, Medical Center—University of Freiburg, Faculty of Medicine, University of Freiburg, Freiburg, Germany; ^9^Institute of Neuropathology, University Hospital Zurich, Zurich, Switzerland

**Keywords:** axon growth, cell adhesion molecule, neuronal connectivity, entorhinal cortex, hippocampus, cognition, seizures, psychiatric disorders

## Abstract

Neuronal growth regulator 1 (NEGR1), a member of the immunoglobulin superfamily cell adhesion molecule subgroup IgLON, has been implicated in neuronal growth and connectivity. In addition, genetic variants in or near the NEGR1 locus have been associated with obesity and more recently with learning difficulties, intellectual disability and psychiatric disorders. However, experimental evidence is lacking to support a possible link between NEGR1, neuronal growth and behavioral abnormalities. Initial expression analysis of NEGR1 mRNA in C57Bl/6 wildtype (WT) mice by *in situ* hybridization demonstrated marked expression in the entorhinal cortex (EC) and dentate granule cells. In co-cultures of cortical neurons and NSC-34 cells overexpressing NEGR1, neurite growth of cortical neurons was enhanced and distal axons occupied an increased area of cells overexpressing NEGR1. Conversely, in organotypic slice co-cultures, *Negr1*-knockout (KO) hippocampus was less permissive for axons grown from EC of β-actin-enhanced green fluorescent protein (EGFP) mice compared to WT hippocampus. Neuroanatomical analysis revealed abnormalities of EC axons in the hippocampal dentate gyrus (DG) of *Negr1*-KO mice including increased numbers of axonal projections to the hilus. Neurotransmitter receptor ligand binding densities, a proxy of functional neurotransmitter receptor abundance, did not show differences in the DG of *Negr1*-KO mice but altered ligand binding densities to NMDA receptor and muscarinic acetylcholine receptors M1 and M2 were found in CA1 and CA3. Activity behavior, anxiety-like behavior and sensorimotor gating were not different between genotypes. However, *Negr1*-KO mice exhibited impaired social behavior compared to WT littermates. Moreover, *Negr1*-KO mice showed reversal learning deficits in the Morris water maze and increased susceptibility to pentylenetetrazol (PTZ)-induced seizures. Thus, our results from neuronal growth assays, neuroanatomical analyses and behavioral assessments provide first evidence that deficiency of the psychiatric disease-associated *Negr1* gene may affect neuronal growth and behavior. These findings might be relevant to further evaluate the role of NEGR1 in cognitive and psychiatric disorders.

## Introduction

Neuronal growth regulator 1 (NEGR1) belongs to the IgLON subgroup of the immunoglobulin superfamily (IgSF) which consists in mammals of five members, IgLON1–5, also termed OBCAM/OPCML (*opioid binding cell adhesion molecule*, IgLON1), Neurotrimin (IgLON2), LAMP/LSAMP (*limbic system associated membrane protein*, IgLON3) and NEGR1 (IgLON4), also known as Kilon or Neurotractin, and IgLON5 (Pimenta et al., 1995; Struyk et al., 1995; Funatsu et al., 1999; Marg et al., 1999; Schäfer et al., 2005; Sabater et al., 2014). Previous studies associated IgLON subgroup members with a wide spectrum of psychiatric disorders. For example, polymorphisms in the human LSAMP gene have been associated with major depressive disorder (Koido et al., 2012) and schizophrenia (Koido et al., 2014). Gene variants in a regulatory region upstream of the NEGR1 gene have been associated with major depressive disorder (Hyde et al., 2016) but also with low white matter integrity (Dennis et al., 2014) and intelligence (Sniekers et al., 2017). Neuropsychiatric problems and learning difficulties were further reported in two siblings with a mono-allelic microdeletion of chromosome 1p31.1 involving only the NEGR1 gene (Genovese et al., 2015). Interestingly, increased cerebrospinal fluid protein levels of NEGR1 were found in major depressive disorder and bipolar disease but not schizophrenia compared to controls (Maccarrone et al., 2013). Other neurological phenotypes possibly related to altered NEGR1 function comprise intellectual disability and language impairment (Tassano et al., 2015), dyslexia (Veerappa et al., 2013) and juvenile myoclonic epilepsy (Naseer et al., 2017).

The biological function of NEGR1 is still elusive but *in vitro* studies suggest a role for neuronal growth and connectivity. It has been shown that IgLON proteins promote neurite growth and may act as attractive or repulsive factors in axonal growth and pathfinding (Pimenta et al., 1995; Mann et al., 1998; Marg et al., 1999; Eagleson et al., 2003; Reed et al., 2004; Sanz et al., 2017). In the hippocampus, IgLON proteins are developmentally up-regulated (Miyata et al., 2003; Schäfer et al., 2005), gene expression is confined to hippocampal subfields (Struyk et al., 1995; Bräuer et al., 2000) and IgLON overexpression in cultured hippocampal neurons modulates synaptogenesis (Hashimoto et al., 2008, 2009). These observations suggested a role of IgLONs in hippocampal circuit formation which has been supported by the findings that LSAMP-deficient mice exhibit deficits in spatial memory acquisition and poorly sustained long-term potentiation in the hippocampus (Qiu et al., 2010). More recently, NEGR1 was shown to augment neuronal arborization and spine density (Pischedda et al., 2014; Pischedda and Piccoli, 2015) and, together with other IgLON members, to serve as a substrate for neurite outgrowth of cortical neurons (Sanz et al., 2015).

The *in vitro* function of NEGR1 together with results from gene associations studies in cognitive and psychiatric disorders, prompted us to examine the role of NEGR1 in neuronal growth and behavior in *Negr1*-deficient mice. We performed functional analysis of NEGR1 in cell and organotypic slice co-culture assays, studied neuroanatomy using DiI tracing of entorhinal axons and determined ligand binding densities of functional neurotransmitter receptors. Finally, we subjected *Negr1*-KO mice to various behavioral tasks to evaluate overall activity, anxiety, sensorimotor gating, social interaction, cognitive performance and seizure susceptibility.

## Materials and Methods

### Reagents

The following antibodies were used for immunocytochemistry (ICC) or immunohistochemistry (IHC): rabbit anti-calbindin (Swant, IHC, dilution 1:5000), mouse anti-GFP (Millipore, mab3580, IHC, 1:500), mouse anti-synaptobrevin 2 (SYB, Synaptic Systems, clone 69.1, ICC, 1:1000), mouse anti-βIII-tubulin (Covance, TUJ1, ICC, 1:2000), Alexa Fluor 488 and Alexa Fluor 568-conjugated secondary antibodies (Life Technologies, ICC, IHC, 1:1000). The neuronal tracer DiI, cell culture medium and supplements were purchased from Life Technologies. Unless otherwise specified laboratory reagents were purchased from Roth or Sigma-Aldrich.

### Animals

Mice were maintained in the animal facilities at Universities of Freiburg, Mainz and Tartu used in accordance with principles of good laboratory animal care and in approval by local authorities (Regierungspräsidium Freiburg, protocol G-08/96; Landesuntersuchungsamt Rheinland-Pfalz, protocol G13-1-018; permit No. 29 from the Estonian National Board of Animal Experiments). Transgenic mice expressing *enhanced green fluorescent protein* (EGFP) under control of chicken β-actin promoter (C57Bl/6-TgCAG-EGFP1Osb/J) were purchased from Jackson Laboratory. Experiments including *Negr1*-KO mice were performed with 2–3 months-old male offspring from mice heterozygous for *Negr1* (*Negr1*^+/–^) and designated as *Negr1*-WT mice (*Negr1*^+/+^) or *Negr1*-KO mice (*Negr1*^−/−^). Mice were bred on a C57Bl/6 background, except mice tested for social interaction and in the Morris water maze which were 2 month old male F2 hybrids [(129S5/SvEvBrd×C57BL/6) × (129S5/SvEvBrd×C57BL/6)]. This background is used as a standard in the University of Tartu, Institute of Biomedicine and Translational Medicine. Mixed background usually exhibits reliable robust effects due to eliminating most of the strain specific effects caused by homozygosity of the loci. Overlapping experiments in Mainz and Tartu, i.e., the tests for locomotor activity (open field test) and anxiety-like behavior (elevated plus-maze) provided similar results and serve as inter-laboratory validation. Generation, genotyping protocol and lack of NEGR1 protein expression in *Negr1*-KO mice were reported before (Lee et al., 2012).

### *In Situ* Hybridization

Brains from inbred C57Bl/6 mice (Charles River) were dissected at postnatal day 1 (P1), at P8, P15, and at P60 (*n* ≥ 3 per stage) processed for *in situ* hybridization essentially as described using digoxigenin (DIG)-labeled cRNA probes (Heinrich et al., 2006). Sense and anti-sense probes were generated against full length murine Negr1 cDNA (Schäfer et al., 2005) and murine Reelin cDNA (Heinrich et al., 2006). *In situ* hybridization of sections from *Negr1*-KO mice did not show any signal with sense or antisense probes. Microscopy was performed with an AxioImager (ZEN software; Zeiss).

### Cell Culture, Transfection and Immunocytochemistry

NSC-34 cells (Cashman et al., 1992) were cultured and transfected using expression plasmids pcDNA3-Negr1 and pEGFP-C1 (Clontech) in 24-well plates as described (Lee et al., 2012). Primary cortical neurons, prepared from embryonic day 18 (E18) mouse embryos. Sixteen hours after NSC-34 cell transfection, 50,000 neurons were seeded per well onto transfected confluent NSC-34 cell cultures, co-cultured for 2 days *in vitro* (DIV), processed for βIII-tubulin immunostaining and the longest neurite of individual neurons was measured (Lee et al., 2012). Axon attraction was assayed using co-cultivation of NSC34 cells transfected with pcDNA3-Negr1 and/or pEGFP-C1 and primary cortical neurons for 2 DIV. Primary neurons were pre-cultivated for 5 DIV to allow axon differentiation before transfected NSC-34 were added. Distal axon segments were immunostained using primary antibodies specific to SYB followed by secondary Alexa Fluor 568-conjugated secondary antibodies. EGFP-positive cells were imaged by identical acquisition parameters and investigators were blind to the experimental conditions. For image analysis, EGFP-positive cells were delineated and the relative area occupied by SYB-immunolabeled axons was calculated as an area ratio using ImageJ software (NIH Image).

### Organotypic Co-cultures

Organotypic co-cultures were prepared and cultivated essentially as described (Brinks et al., 2004; Lacour et al., 2007). Briefly, 400 μm thick hippocampal slices were prepared from P1–P3 *Negr1*-WT and *Negr1*-KO mice using a tissue chopper. Entorhinal cortex (EC) slices (400 μm) were prepared from P3 transgenic β-actin-EGFP mice and accurately aligned to hippocampal explants. After 2 weeks of cultivation, co-cultures were fixed for 2 h in 4% paraformaldehyde (PFA), washed in PBS, re-sliced by vibratome sectioning (50 μm) and processed as described below.

### Immunostaining of Organotypic Slice Cultures and DiI Tracing of Entorhinal Axons

Immunostaining of organotypic co-cultures was performed according to standard procedures using antibodies specific to calbindin to visualize granule cells and GFP to enhance the genetically-driven GFP-signal, respectively. Double-immunofluorescent images were acquired using an inverted light microscope (Olympus BX60), equipped with a monochrome digital camera (Leica, DFC350FX). Exclusion criteria for further analysis were dispersion of granule cell and pyramidal cell layers or absence of accurate mossy fiber projections. A total of 78 slice co-cultures fulfilling these criteria were analyzed wildtype (WT: *n* = 41; KO: *n* = 37). Line scans were applied to provide densitometric data of EGFP fluorescence (as a measure for entorhinal axons) along the vertical axis from proximal to distal dendrites of calbindin-immunolabeled granule cells. One line scan was applied per slice co-culture and one data point per micrometer (μm) dendrite length was collected over a total length of 134 μm using the “plot profile” function of ImageJ (NIH Image). Values from each line scan were normalized to the first data point recorded at the outer calbindin-positive granule cells which were devoid of EGFP fluorescence (set to 1).

For DiI tracing experiments, mice were anesthetized and transcardially perfused with PBS followed by 4% PFA/PBS dissolved in PBS. Dissected brains were post-fixed overnight in 4% PFA and transversal tissue blocks were prepared for retrograde DiI tracing. Small crystals of the fluorescent tracer DiI (Molecular probes) were placed under microscopic control onto the upper layers of the EC using a glass micropipette. Tissue blocks were stored in 4% PFA for 3 weeks at room temperature to allow sufficient diffusion of the tracer along entorhinal fibers. Tissue blocks were sectioned transversally at 80 μm using a vibratome, counterstained with DAPI (4′,6-diamidino-2-phenylindole, Invitrogen), coverslipped and immediately imaged using an inverted light microscope (Olympus BX60), equipped with a monochrome digital camera (Leica, DFC350FX). Images of tissue sections from *Negr1*-WT and *Negr1*-KO mice showing dense DiI-labeled entorhinal projections to the outer molecular layer (oml) were inspected for axons traversing the granule cell layer (gcl; WT = 95 sections, KO = 115 sections, *n* = 7 mice per genotype).

### Neurotransmitter Receptor Autoradiography

Labeling of neurotransmitter receptors was essentially performed as described (Frauenknecht et al., 2014). Briefly, frozen and unfixed brains from 2-month-old *Negr1*-WT and *Negr1*-KO mice (*n* = 8 per genotype) were carefully removed, immediately frozen in isopentane at −20°C, and stored at −80°C until use. Brains were then serially cut at the level of the striatum and at the level of the hippocampus into 20 μm thick coronal cryostat sections. Cutting of slices started for the striatal level at Bregma 1.045 mm and for the hippocampus at Bregma −1.555. From each level 32 subsequent 20 μm thick slices were collected. Slices 3–7 from each level (striatum as well as dorsal hippocampus) were collected and processed for quantitative receptor autoradiography of glutamate receptors NMDAR and AMPAR, the inhibitory GABAergic receptor GABAAR and the muscarinic acetylcholine receptors M1R and M2R. Ligands were purchased from Perkin Elmer Inc., Waltham, MA, USA. Labeling and incubation procedures were performed according to the protocols by Zilles et al. (2000, 2004) as previously described (Frauenknecht et al., 2013). Densitometric analyses of autoradiographic films were performed, as described previously (Frauenknecht et al., 2009; Diederich et al., 2012). Briefly, autoradiographies were scanned under equal lighting conditions (CoolSNAP camera; Roper Scientific, Photometrics CoolSNAP™ see Ottobrunn/Munich Germany) and were digitized (MCID image analysis system, Imaging Research Inc., St. Catharines, ON, Canada). Receptor binding densities were calculated for the hippocampus subregions CA1, CA3 and dentate gyrus (DG) with their dendritic, pyramidal, molecular and granular layers, according to the stereotaxic mouse brain atlas by Paxinos and Franklin (2001) as described (Frauenknecht et al., 2014). The investigator, blind to genotype of samples, analyzed ligand binding densities by calculating mean concentration values for each ligand and region. Co-exposed microscales were used to calculate the relationship between gray values in the autoradiographs and concentrations of radioactivity, and values were normalized to control levels.

### Open Field Test

For the open field test, mice (*n* = 11 per genotype) were placed into the center of a plastic cylinder (120 cm in diameter and 40 cm high) in a lit room (120 lx). Behavior was recorded for 7 min starting from the first wall approach. Distance traveled, velocity, and time spent in the center zone were measured using a video tracking system (Olympus).

### Elevated Plus Maze Test

The apparatus consists of open and closed arms, crossed in the middle perpendicularly to each other, and a center area. An illumination of 120 lx was applied. Mice were placed in the central platform, facing an open arm of the maze. Subsequently, the behavior was recorded for 5 min by an overhead video camera and a PC equipped with “EthoVision XT 8.0” software (Version 8.0, Noldus, 2010) to calculate the time spent in opened or closed arms, number of arm visits as well as distance traveled and the velocity. The number of entries into the open arms and the time spent in the open arms are used as indices of open space-induced anxiety in mice (*n* = 11 per genotype).

### Prepulse Inhibition Test

The prepulse inhibition (PPI) test was performed with an acoustic startle reflex measurement system (Med Associates, USA). A test session began by placing a mouse (*n* = 11 per genotype) in the experiment chamber (9 × 4 × 4 cm) where it was left undisturbed for 5 min. The duration of white noise (continuous throughout the session), used as the startle stimulus, was 40 ms for all test types. The startle response was recorded for 140 ms starting with the onset of the prepulse stimulus. The background noise level in each chamber was 65 dB. The peak startle amplitude recorded during the 140-ms time frame was used as readout. A test session consisted of four types of test (i.e., a test of the starting stimulus only, and three types of PPI study). The intensity of the acoustic starting stimulus was 120 dB. The prepulse sound was triggered 100 ms before the starting stimulus, and the stimulation intensity was 75, 80 and 85 dB. Each trial type consisted of 10 individual experiments. Experiments of all kinds were carried out in a random order. The time intervals were varied between 10 s and 20 s. Amplitudes were determined as described (Radyushkin et al., 2009) and the PPI was calculated as the percentage decline in the startle response:

$$\text{prepulse inhibition }[\%]\;=\;100\;-\;\left[\left(\frac{\left(\text{shock amplitude after prepulse and pulse}\right)}{\left(\text{shock aplitude after pulse only}\right)}\right)\;\times \;100\right]$$

### Three-Chamber Sociability Test

Sociability behavior of mice (*n* = 20 per genotype) were assessed by means of an automated three-chamber apparatus. Briefly, the test was performed in three stages, each lasting 10 min, as follows: (1) first habituation session consisting of a 5-min habituation period in an isolated cage and a 5-min habituation in the center chamber; (2) the second 10-min habituation session where the doors were open; the mice were placed in the center and allowed to freely explore all the three chambers; and (3) sociability test where the test mouse was isolated to the center chamber (Chamber 2), while a gender/age-matched stranger (Stranger 1) was placed inside a social enclosure (Noldus) in either Chamber 1 or 3. The other chamber contained an empty social enclosure. The doors were opened, and the test mouse was allowed to freely explore all the chambers. All stranger mice were males at the same age and habituated to the apparatus during the previous day (30-min habituation three times). The positions of the object and Stranger 1 were alternated between tests to prevent side preference. The tests were video-recorded and time spent in each chamber during stages 2 and 3 were measured by the tracking software (EthoVision XT 8.5, Noldus Technology).

### Social Dominance Tube Test

The test apparatus was adapted from previous work (Lijam et al., 1997; Koh et al., 2008) with some modifications in the experimental design. Two waiting chambers, sized 10 × 10 × 10 cm, were connected by a 30 cm clear plexiglas tube (3 cm diameter). One *Negr1*-WT mouse and one *Negr1*-KO mouse were placed in the waiting chambers at the opposite ends of the tube and were thereafter simultaneously released into the tube. The mouse that remained in the tube, while its opponent completely backed out from the tube, was declared “winner”. The winner was given a score “1” and the loser a score “0”. Each trial lasted a maximum of 5 min and an even score “0.5” was counted when both opponents remained into the tube. During testing, the room was dimly lit with diffuse white light (20 lx). Each mouse was tested three times with three different weight-matched mice of the opposite genotype (*n* = 20 per genotype).

### Morris Water Maze

The water maze consisted of a circular pool (150 cm in diameter), escape platform (16 cm in diameter), video camera and computer with EthoVision software (Noldus Technology). The pool was filled with tap water (22°C) that was made opaque by adding a small amount of non-toxic white putty. The escape platform was positioned in the center of the Southwest quadrant (Q2), 20 cm from the wall. The water level was 1 cm above the platform, making it invisible. Each trial, the animals were put into the water, facing the wall, at pseudo-randomly assigned starting positions (East, North, South or West). The acquisition phase of the experiment consisted of a series of 16 training trials, lasting up to 60 s each (four trials per day for four consecutive days, inter-trial interval *ca*. 1 h). Mice were allowed to search for the platform for a maximum of 60 s at which time the mice were gently guided to the platform by means of a metal sieve. Mice remained on the platform for 15 s. Furniture around the maze served as visual cues. During testing, the room was dimly lit with diffuse white light (20 lx). Distance traveled during the trial, latency to find the submerged platform and swim velocity were registered. We used average value per day, which was obtained by collapsing data on five trials for each animal. On day 4, 1 h after the last training trial, the platform was removed for a probe trial. Mice were put into the water in the Northeast position (Q4) and were allowed to swim for 60 s. Time spent in all four quadrants (Q1, Q2, Q3, Q4) was measured, with time spent in the target quadrant (Q2) where the platform had been located serving as indicator of spatial memory. On days 5 and 6 the platform was positioned in the Southwest position (Q3) and reversal training was performed (four trials per day). On day 6, 1 h after the last training trial, the platform was removed for a similar probe trial as above. A total of 27 mice were tested (*Negr1*-WT: *n* = 13; *Negr1* KO: *n* = 14).

### PTZ Seizure Susceptibility Test

Seizure susceptibility in mice (*Negr1*-WT: *n* = 13, *Negr1*-KO: *n* = 14) was studied after single administration of pentylenetetrazol (PTZ; s.c. 50 mg/kg body weight) and monitored for 30 min using a video camera. The seizure severity was scored essentially as described (Ferraro et al., 1999; Pöttker et al., 2017).

### Statistics

Data were analyzed using GraphPad Prims 6 (GraphPad Software, San Diego, CA, USA). Data distribution was tested by Shapiro-Wilk normality test and statistical significance was evaluated by the *t*-test for parametric and by Mann-Whitney *U-test* for non-parametric data or two-way analysis for variance (ANOVA), as specified in figure legends. All data were expressed as mean ± standard error of mean (SEM).

## Results

### *NEGR1* mRNA Is Markedly Expressed in the Entorhino-hippocampal Formation

**Figure 1:**
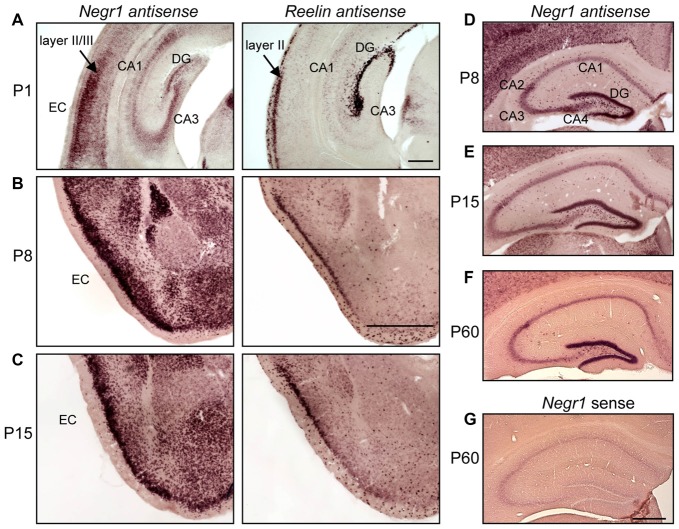
Neuronal growth regulator 1 (NEGR1) mRNA expression in the developing entorhino-hippocampal formation. **(A–C)** *In situ* hybridization for *Negr1* and *Reelin* in adjacent brain sections at several postnatal stages showing NEGR1 mRNA expression in the hippocampus and entorhinal cortex (EC) at P1 **(A)**, and in layers II/III of the EC at P8 **(B)** and P15 **(C)**. *Reelin* was detected in adjacent sections to identify the layer II/III of the EC. Scale: 200 μm **(A)**; 500 μm **(B,C)**. **(D–F)** *In situ* hybridization for *Negr1* at P8 **(D)**, P15 **(E)** and P60 **(F)** shows expression in cornu ammonis areas CA1–CA4 and granule cells of the dentate gyrus (DG). **(G)** *Negr1* sense control is devoid of any labeling. Scale: 500 μm **(D–G)**.

Initially, NEGR1 mRNA expression analysis was performed at different developmental stages by *in situ* hybridization. Marked expression of NEGR1 mRNA was found in the EC and dentate granule cells of C57Bl/6 mice. At postnatal day P1 the antisense probe revealed NEGR1 mRNA expression in the hippocampal proper, the DG as well as in the EC which was identified using an antisense probe for the EC layer II marker *Reelin* in adjacent sections (Figure 1A). Strong expression of *Negr1* was observed at P8, P15 and P60 in layers II/III of the EC (Figures 1B,C, P60 not shown). In the hippocampus at P8, P15 and P60, *Negr1* expression is highly concentrated in the DG. In CA1 and CA3 the signals appeared concentrated in interneurons rather than pyramidal neurons. *Negr1* expression was high at P8 in CA4 and unchanged in CA2 from P8 to P60 (Figures 1D–F). *Negr1* sense probe did not show any signal (Figure 1G).

### *NEGR1* Promotes Neurite Growth and Is Permissive for Axon Growth of Primary Cortical Neurons

**Figure 2:**
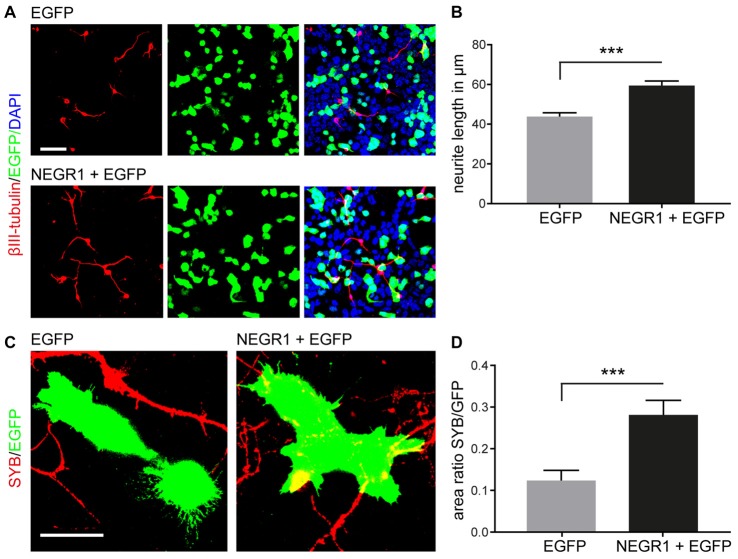
NEGR1 promotes neurite growth and is permissive for axon growth. **(A)** Co-cultures of primary cortical neurons and NSC-34 cells expressing NEGR1 and/or enhanced green fluorescent protein (EGFP) immunostained by βIII-tubulin/anti-EGFP antibodies after 2 days of co-cultivation. Cell nuclei were stained by DAPI. **(B)** NEGR1 expressed by NSC-34 cells promotes neurite growth of co-cultured cortical neurons compared to control (EGFP alone). Mean neurite length per neuron is shown (total number of neurites: NEGR1 + EGFP = 469; EGFP alone = 399, pooled from two independent experiments). **(C)** Images showing co-cultures of NSC-34 cells expressing NEGR1 and/or EGFP which have been added to primary cortical neurons after 5 days *in vitro* (DIV) and cultivated for further 2 DIV. Cortical axons were immunostained by anti-synaptobrevin 2 (SYB). **(D)** The SYB/EGFP area ratio, resembling the area occupied by distal axons on EGFP-positive NSC-34 cell bodies is increased by NEGR1 expression (total number of analyzed cells: NEGR1 + EGFP = 48; EGFP alone = 48, pooled from two independent experiments). ***p < 0.001, Mann Whitney U test. Scales: 50 μm **(A)**; 20 μm **(C)**.

The mRNA expression pattern of *Negr1* together with the capability of NEGR1 protein to promote cell-cell adhesion and neurite growth (Schäfer et al., 2005; Lee et al., 2012) suggested that NEGR1 may contribute to growth of entorhino-hippocampal projections. We therefore tested whether NEGR1 stimulates neurite growth and is permissive for axon growth of cortical neurons. Neurite growth was studied following overexpression of NEGR1 together with EGFP, or EGFP alone, in NSC-34 cells which served as a biological substrate for co-cultured primary cortical neurons (Figure 2A). Two days after co-cultivation, neurons were immunostained for the neuronal marker βIII-tubulin. We found that NEGR1 overexpression in NSC-34 cells stimulates neurite growth of co-cultured cortical neurons (Figure 2B). We next investigated whether NEGR1 is permissive for axon growth. Following transfection of NSC-34 cells with NEGR1 and/or EGFP expression constructs, cells were co-cultured for 2 days with differentiated primary cortical neurons which have been pre-cultured for 5 days. Immunostaining with antibodies specific for the presynaptic and distal axon marker Synaptobrevin (SYB)-2 revealed that axons occupied an increased area of NEGR1/EGFP-expressing NSC-34 cells compared to control cells expressing EGFP alone, suggesting a preference of distal axons for cells expressing NEGR1 (Figures 2C,D).

### Entorhinal Axon Growth Is Impaired in the Hippocampus of *Negr1*-KO Mice

**Figure 3:**
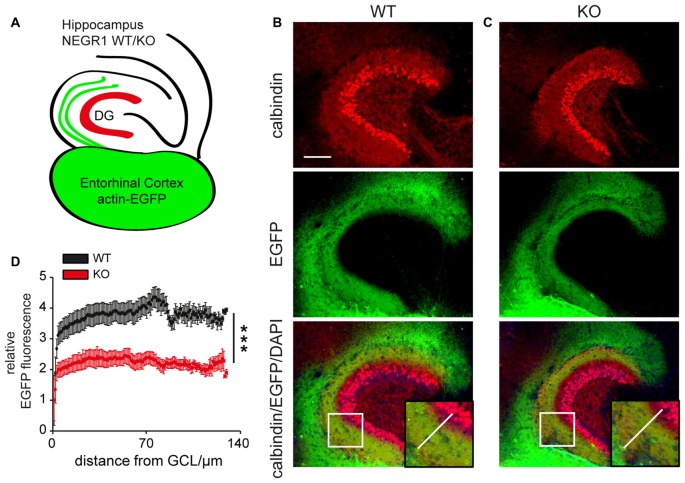
Entorhinal axon growth is impaired in the *Negr1*-deficient hippocampus. **(A)** Scheme illustrating the organotypic co-culture assay. **(B,C)** Anti-EGFP/anti-calbindin/DAPI co-labeling shows ingrowth of EGFP-expressing entorhinal axons into the DG of *Negr1*-wildtype (WT) mice **(B)** or *Negr1*-knockout (KO) mice. (**B,C**, insert) Images were processed for line scan analysis across the granule cell layer (GCL) with respect to calbindin-immunostaining as depicted. **(D)** Line scan analysis of GCL from WT (*n* = 41) and KO mice (*n* = 37) shows a decreased and less gradual entorhinal axon ingrowth in *Negr1*-KO mice. ***p < 0.001, Mann Whitney U test. Scale: 100 μm **(B)**.

To examine whether NEGR1 affects growth of entorhinal cortical axons in the hippocampus, we used organotypic brain tissue co-cultures (Lacour et al., 2007). To unambiguously identify entorhinal axons, we co-cultured entorhinal cortices from β-actin-EGFP transgenic mice with hippocampal slices from *Negr1*-KO mice or *Negr1*-WT littermates (Figure 3A). After 2 weeks of cultivation, co-cultures were sliced with a vibratome and processed for double-immunostaining with antibodies specific to GFP or calbindin to visualize granule cells and their dendrites which demarcate the molecular layer of the DG (Figures 3B,C). EGFP fluorescence intensities were determined by line scans along the vertical axis from the cell bodies of outer calbindin-positive granule cells until their dendritic endpoints aligning the hippocampal fissure (Figures 3B,C, inserts). In hippocampal slices from *Negr1-WT* mice, EGFP-fluorescence in the molecular layer showed a moderately graded distribution along the vertical axis and a fourfold increased intensity compared to the non-invaded granule cell layer. In contrast, a less pronounced graded distribution and an overall reduced EGFP-fluorescence was found in the molecular layer of the hippocampus from *Negr1*-KO mice (Figure 3D).

### *Negr1*-KO Mice Exhibit Abnormal Entorhinal Axon Projections in the Hippocampus

**Figure 4:**
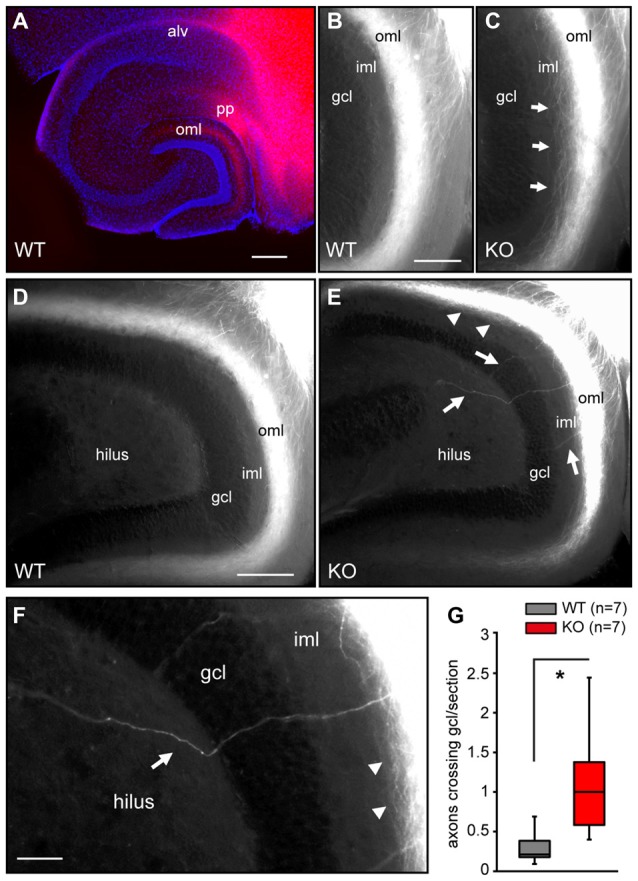
*Negr1*-KO mice exhibit abnormal entorhinal axon projections in the hippocampus.** (A)** Anterograde DiI-labeling in a transversal tissue block from *Negr1*-WT mice showing entorhinal axon projections (alv, pp). Note labeling of entorhinal axons in the oml of the DG. **(B,C)** DiI-labeled entorhinal axons in the DG. Axons appear less fasciculated in *Negr1*-KO mice (arrows) compared to *Negr1*-WT mice. **(D,E)** DiI-labeled entorhinal axons invade and eventually cross the iml and gcl in *Negr1*-KO mice (arrows). **(F)** Detail magnification of **(E)** showing DiI-labeled defasciculated axons (arrowheads) and a single entorhinal axon crossing the gcl (arrow). **(G)** Box plot showing the distribution of DiI-labeled axons crossing the gcl per section. WT (*n* = 7, 115 tissue sections) and *Negr1*-KO mice (*n* = 7, 95 tissue sections). *p < 0.05, Mann Whitney U test. Scales: 200 μm **(A)**; 50 μm **(B,C)**, 100 μm **(D,E)**; 25 μm **(F)**. Abbreviations: alv, alvear path; pp, perforant path; gcl, granule cell layer; iml, inner molecular layer; oml, outer molecular layer.

The results obtained in the organotypic slice co-culture model suggested that hippocampal NEGR1 may instruct growth and possibly targeting of EC axons. To trace entorhinal axons in the brain, DiI-crystals were positioned into the upper layers of the EC in PFA-fixed transversal tissue blocks. Three weeks later, diffusion of the neuronal tracer was observed in the major entorhino-hippocampal pathways, the alvear path (alv) and the perforant path (pp; Figure 4A). However, DiI-labeled axons appeared less fasciculated in the molecular layer of the DG in *Negr1*-KO compared to WT littermates (Figures 4B,C). We further observed particularly in *Negr1*-KO mice, DiI-labeled axons crossing the inner molecular layer (iml) and approaching or even traversing beyond the gcl into the hilar region (Figures 4D–F). Indeed, the number of DiI-labeled axons traversing the gcl was significantly increased in *Negr1*-KO mice compared to *Negr1*-WT mice (Figure 4G).

### *Negr1*-KO Mice Show Subtle Changes in Neurotransmitter Receptor Ligand Binding in Distinct Hippocampal Subfields

**Figure 5:**
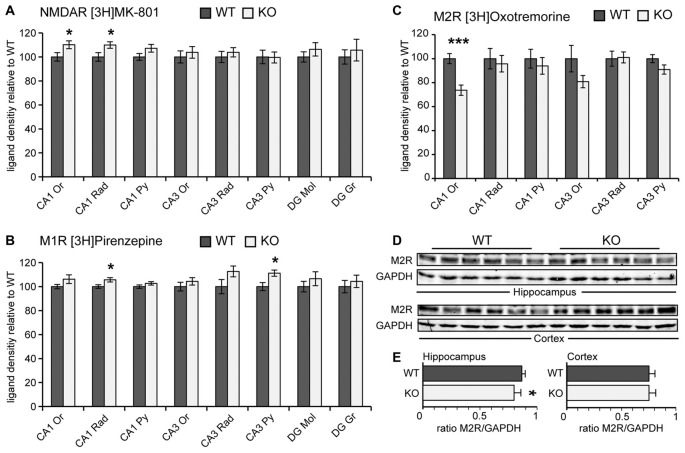
*Negr1*-KO mice show alterations in neurotransmitter receptor ligand binding in distinct hippocampal subfields.** (A–C)** Relative binding densities of [3H]MK-801 to NMDAR **(A)**, [3H] Pirenzepine to M1R **(B)**, and [3H] Oxotremorine to M2R **(C)**. Please note that ligand binding densities for M2 AChR in the DG were in the same range of unspecific binding and have been omitted **(C)**. Values are expressed as mean value in % ± standard error of mean (SEM), *n* = 8 for each genotype. Parametric data were analyzed by Student’s *t*-test, non-parametric data by Mann Whitney U test. **(D)** Western Blot showing M2R protein expression in total hippocampus lysates. GAPDH was used as a reference protein. **(E)** Histograms showing slightly reduced M2R/GAPDH protein band intensity ratio in hippocampus but not in cortex samples from *Negr1*-KO mice compared to WT (*n* = 6 per genotype). *p < 0.05, ***p < 0.001, Student’s *t*-test. Abbreviations: CA, cornu ammonis, DG, dentate gyrus, Or, stratum oriens, Rad, stratum radiatum, Py, stratum pyramidale, Mol, stratum moleculare, Gr, stratum granulare.

The abnormalities in entorhinal fiber projections in *Negr1*-KO mice prompted us to determine the distribution of functional neurotransmitter receptors in the hippocampus. To this end, we performed quantitative receptor autoradiography to analyze ligand binding densities of NMDAR, AMPAR, GABAAR, M1R and M2R in hippocampal subfields (Frauenknecht et al., 2014). AMPA receptor and GABAA receptor ligand binding were unchanged compared to WT (data not shown). *Negr1*-KO mice also did not exhibited altered ligand binding densities in the molecular layer or the gcl of the DG. Instead of changes in the DG, NMDAR ligand binding was significantly increased both in stratum oriens and stratum radiatum in the CA1 region of *Negr1*-KO mice (Figure 5A). Contrasting ligand binding densities were found for mACh M1 and M2 type receptors. While M1R ligand binding was slightly increased in CA1 stratum radiatum and CA3 pyramidal cell layer (Figure 5B), a significantly decreased MR2 ligand binding was evident in CA1 stratum oriens (Figure 5C). In the DG, ligand binding intensities for M2R were below the detection limit (data not shown). As ligand binding densities provide information on functional neurotransmitter receptor abundance (Müller et al., 2010), but not on expression levels, we determined total M2R protein levels by western blot. We found that M2R protein levels were slightly reduced in the hippocampus but not in the cortex from *Negr1*-KO mice compared to WT (Figures 5D,E). These findings indicate subtle changes in the abundance of functional neurotransmitter receptors in distinct hippocampal subfields in *Negr1*-KO mice.

### *Negr1*-deficiency Does Not Affect Activity, Anxiety-like Behavior and Sensorimotor Gating

**Figure 6:**
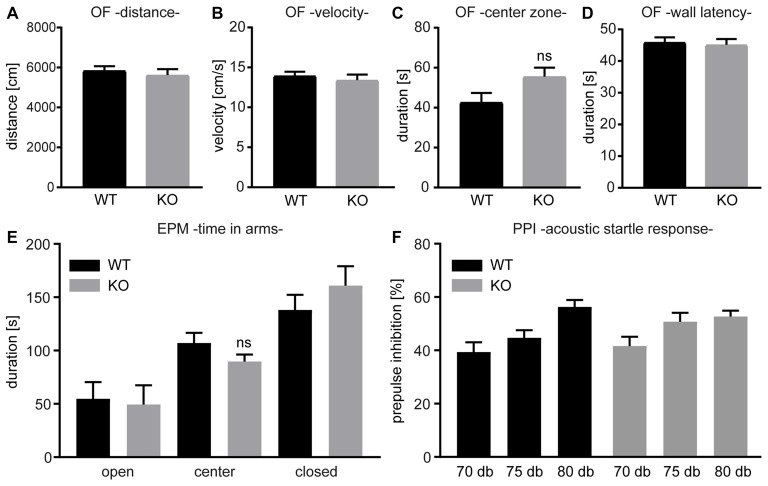
Activity, anxiety-like behavior and sensorimotor gating are normal in *Negr1*-deficient mice.** (A–D)** The open field test (OF) was used to evaluate overall activity and anxiety-like parameters such as distance traveled **(A)**, velocity **(B)** time spent in center zone (**C**, ns = not significant, *p* = 0.059, Student’s *t*-test) or in wall latency **(D)**. **(E)** The elevated plus maze test (EPM) was applied as an additional test to assess anxiety-like behavior. **(F)** The prepulse inhibition (PPI) test was performed with an acoustic startle reflex measurement system. No statistical differences were noted between *Negr1*-WT and *Negr1*-KO mice in the OF, EPM, or PPI (*n* = 11 per genotype).

To characterize the behavior, we subjected cohorts of *Negr1*-KO mice and *Negr1*-WT littermate mice to behavioral tasks. Overall activity and anxiety-like behavior was examined in the open field test (Figures 6A–D) and the elevated plus maze test (Figure 6E). However, mice of both genotypes performed normally and *Negr1*-KO mice did not exhibit overt abnormalities. Next, we assessed sensorimotor gating using PPI test and measured the acoustic startle response (Figure 6F). Again, both *Negr1*-KO mice and *Negr1*-WT behaved normally. These results indicate that, *Negr1*-deficiency does not affect activity, anxiety-like behavior and sensorimotor gating.

### *Negr1*-deficiency Impairs Social Behavior

**Figure 7:**
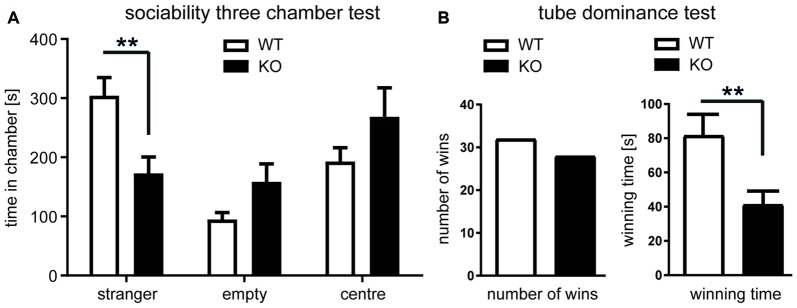
*Negr1*-deficiency impairs social behavior.** (A)** Three-chamber sociability test. The time spent in each chamber was determined for *Negr1*-WT and *Negr1*-KO mice (*n* = 20 per genotype). *Negr1*-KO mice interacted significantly less time with stranger mice compared to *Negr1*-WT mice. **(B)** Tube dominance test. Male mice with opposite genotype were paired for testing (*n* = 20 per genotype) and the number of wins and the latency to win was determined. While the number of wins was similar in both genotypes, the average latency to win was significantly shorter in *Negr1-KO* mice (right), indicative of altered pattern of social interaction; **p < 0.01, Student’s *t*-test.

We next studied social interaction as a behavioral marker for neurodevelopmental disorders (Moy et al., 2004). To evaluate voluntary social interaction, we performed the three-chamber sociability test (Figure 7A). Compared to *Negr1*-WT mice, *Negr1*-KO mice spent significantly less time in the chamber with a stranger mouse and spent proportionally more time in the empty central chamber and the chamber containing an inanimate object (Figure 7A). To examine social approach-avoidance behavior and dominance, mice were subjected to the tube dominance test. While the number of wins achieved by *Negr1*-WT and *Negr1*-KO mice was almost equal, the winning time of *Negr1*-KO mice was significantly shorter (Figure 7B). This result suggests lack of motivation of *Negr1*-KO mice to compete/interact with the other mouse at the later stages of the encounter, leading to withdrawal instead, and possibly compensated by more dominant/aggressive approach of *Negr1*-KO mice in earlier stages of the encounter. Together, these results indicate that *Negr1*-deficiency impairs social behavior.

### *Negr1*-KO Mice Show Deficits in Reversal Learning Behavior

**Figure 8:**
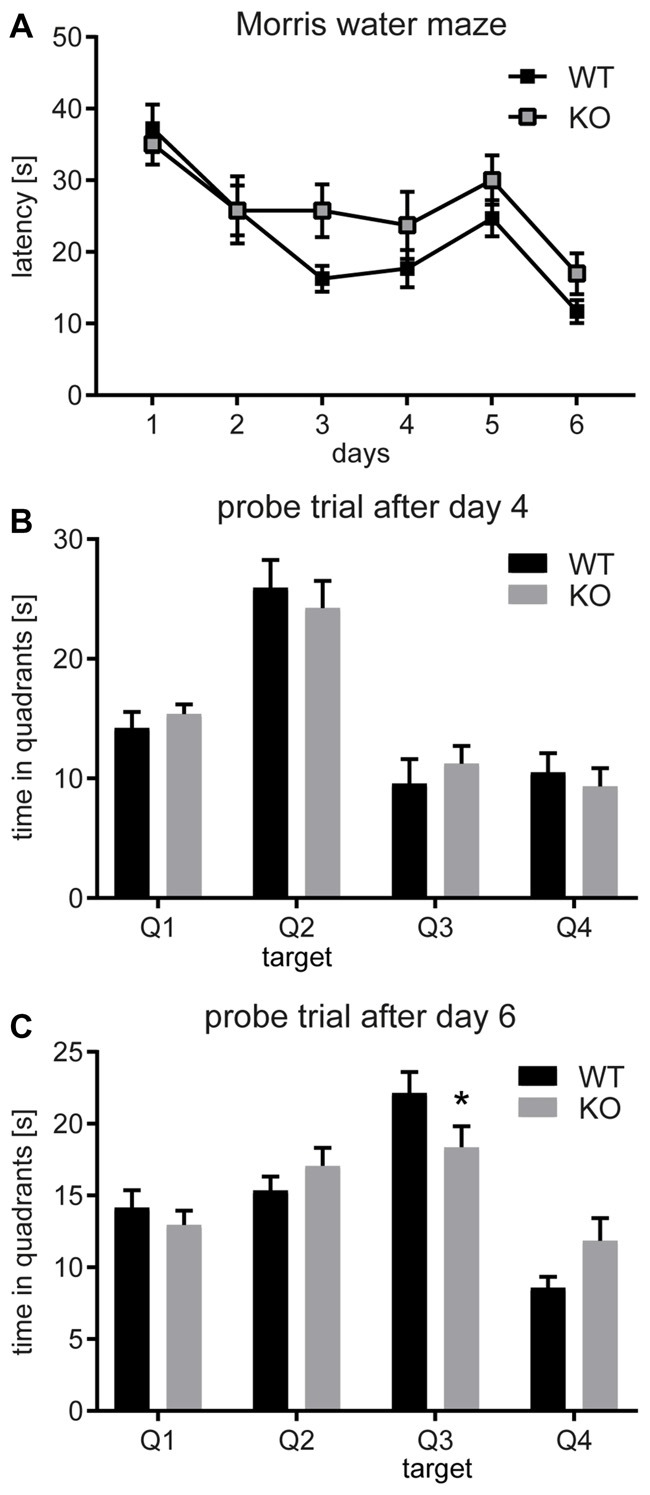
*Negr1*-deficiency causes deficits in reversal learning.** (A)** The learning curve in the Morris water maze showing the time (in seconds) to reach the submerged platform at days 1–4 (initial learning) and days 5–6 (reversal learning). Average values per day, obtained by collapsing data of four trials for each animal, are presented. In **(B,C)** time spent in each of the four quadrants in probe trials after day 4 and day 6, respectively, is presented. In the second probe trial **(C)**, following reversal learning, *Negr1-KO* mice spent significantly less time exploring the target quadrant. A total of 27 mice were tested (*Negr1*-WT: *n* = 13; *Negr1* KO: *n* = 14); *p < 0.05, Student’s *t*-test.

To study the role of NEGR1 in long-term spatial memory, we compared the performance of *Negr1*-WT and *Negr1*-KO mice in the hippocampus-dependent Morris water maze (Morris et al., 1982). *Negr1*-WT mice demonstrated improvement in the time required to find the hidden platform (escape latency) over the first three training days and there was a highly significant improvement from day 1 to day 3 (*p* = 0.00002). *Negr1*-KO mice showed less pronounced improvement from day 1 to day 3 (*p* = 0.058) but performed similar to WT mice at day 4 (Figure 8A). Thus, *Negr1*-KO mice may have lagged in the learning phase. However, no gross learning impairment was evident as in the probe trial after day 4 the performance of both groups was similar (Figure 8B). In contrast, the probe trial performed after completion of 2 days of reversal training at day 6 revealed a statistically significant difference in time spent on scanning the target quadrant with the hidden platform. *Negr1*-KO mice spent significantly less time in the changed target quadrant (Figure 8C). Overall, these results suggest that *Negr1*-KO mice are slower learners than *Negr1*-WT mice and exhibit an impaired relearning capacity.

### *Negr1*-KO Mice Display Increased Susceptibility to PTZ-Induced Seizures

**Figure 9:**
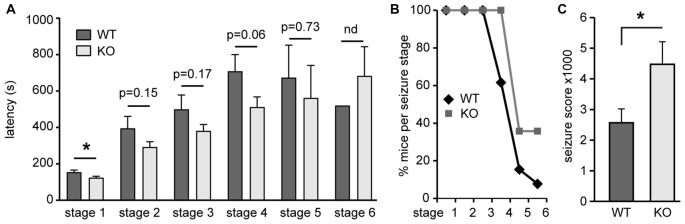
*Negr1*-KO mice display increased susceptibility to induced seizure. **(A)** Relative distribution of mice (WT: *n* = 13, KO: *n* = 14) reaching different seizure stages following single administration of pentylenetetrazol (PTZ; 50 mg/kg/body weight). **(B)** Shown are % mice per seizure stage (WT; KO: *n* = 13; 14 at seizure stage 0). **(C)** Seizure score following single administration of PTZ. *p < 0.05, Mann Whitney U test.

The neuroanatomical abnormalities together with changes in neurotransmitter receptor abundance suggested that *Negr1*-KO mice may exhibit an imbalance in excitation/inhibition. To test this, mouse behavior was monitored for 30 min after single administration of PTZ. *Negr1*-KO mice responded differently after PTZ administration compared to *Negr1*-WT mice (Figures 9A–C). First, *Negr1*-KO mice showed shorter latency to stage 1 (hypoactivity) and a trend towards shorter latency to stage 2 (head nodding), stage 3 (forelimb clonus), and stage 4 (generalized seizures; Figure 9A). Second, the percentage of *Negr1*-KO mice developing generalized seizures (stage 4), tonic seizures (stage 5) or deceased (stage 6) was increased (Figure 9B). Third, seizure score calculation over all seizure stages (Ferraro et al., 1999; Pöttker et al., 2017) revealed significantly increased susceptibility to induced seizures of *Negr1*-KO mice compared to WT mice (Figure 9C).

## Discussion

Abnormalities of brain development and neuronal connectivity likely play a role in the origin of learning and intellectual disabilities as well as psychiatric disorders (McGrath et al., 2006; Bakos et al., 2015). Different classes of synaptic cell adhesion molecules (CAMs) including cadherins (Redies et al., 2012), neurexins and neuroligins (Südhof, 2008), and IgSF-CAMs (Frei and Stoeckli, 2014, 2017; Hortsch et al., 2014) including the IgLON subgroup, have been linked to cognition and its disorders. For example, gene variants of SynCAMs, which possess three extracellular Ig-like domains as IgLON proteins, have been identified as causal or risk factors for intellectual disability and autism spectrum disorders and SynCAM-deficient mice exhibit social and emotional deficits (Frei and Stoeckli, 2017; Gennarini and Furley, 2017). More recently, genetic variants in or near the NEGR1 locus have been associated with cognitive disorders including psychiatric disease, intellectual disability and learning difficulties (Veerappa et al., 2013; Genovese et al., 2015; Tassano et al., 2015; Hyde et al., 2016). These findings prompted us to examine the brain distribution of NEGR1 mRNA, its role in neurite and axon growth and to provide an initial neuroanatomical and behavioral characterization of *Negr1*-KO mice.

Using *in situ* hybridization we demonstrate marked expression of NEGR1 mRNA in layer II/III neurons in the developing EC and granule cells in the DG of C57Bl/6 mice. Entorhinal fibers invade the DG at late embryonic stages and their density increases between P2–P5 in the outermost aspect of the molecular layer (Supèr and Soriano, 1994). At P2–P5 the entorhino-hippocampal afferents further establish morphologically mature synaptic contacts (Borrell et al., 1999). Thus, the spatiotemporal expression of *Negr1* mRNA coincides with maturation of entorhino-hippocampal connectivity. We further showed in cell co-culture assays that NEGR1 promotes neurite growth and is permissive for axon growth of cortical neurons *in trans*, when expressed by co-cultured NSC-34 cells. Our data support recent findings showing a *trans*-activating role of IgLON members in neurite growth (Sanz et al., 2015). Since four members of the IgLON subgroup are expressed in cultured primary cortical neurons (Sanz et al., 2015), and undergo homophilic as well as heterophilic interactions (Gil et al., 1998; Marg et al., 1999; Reed et al., 2004; Lee et al., 2012), cooperative *trans*-interactions of IgLON proteins including NEGR1 might be crucial for growth and differentiation of cortical neurons. It has been further hypothesized that IgLON proteins are important at later stages of brain development rather than for the initiation of neurite outgrowth (Sanz et al., 2015). This hypothesis is compatible with our data obtained from organotypic slice co-cultures which were co-cultured for 2 weeks. Also, the involvement of NEGR1 in axon growth and possibly specification of neuronal connectivity is in line with recent findings on the NEGR1-related IgLON member LSAMP which facilitates reciprocal collection and guidance of dopaminergic afferents to the lateral subnucleus of the habenula in mice (Schmidt et al., 2014).

Our data suggests that NEGR1 contributes to entorhinal axon growth in the hippocampus. First, *Negr1*-KO hippocampus was less permissive compared to *Negr1*-WT hippocampus for the growth of axons from β-actin-EGFP EC. Second, DiI-labeling revealed defasciculated entorhinal axons in the oml in *Negr1*-KO mice but not in WT mice. Third, a significantly increased number of DiI-labeled axons traversing the gcl were observed in *Negr1*-KO mice. These findings suggest a role of NEGR1 in axonal fasciculation and pathfinding as exerted by many neural members of the IgSF-CAMs (Rougon and Hobert, 2003; Kamiguchi, 2007; Barry et al., 2010; Frei et al., 2014). However, similar to other mouse models lacking IgSF-CAMs (Sakurai, 2017), *Negr1*-KO mice displayed a subtle neuroanatomical and behavioral phenotype.

A causal relationship between neuroanatomical and behavioral phenotype of *Negr1*-KO mice has not been investigated in our study and more detailed analyses of axonal pathfinding is required in future studies. Nevertheless, the mild reversal learning deficits in a spatial task and the increased susceptibility to PTZ-induced seizures of *Negr1*-KO mice suggests the involvement of entorhino-hippocampal connectivity, which plays an important role in spatial learning and memory as well as in seizure induction (Kelley and Steward, 1996; Kirkby and Higgins, 1998; Kopniczky et al., 2005, 2006). Also, it appears possible that alterations in the density and distribution of neurotransmitter receptors may contribute to increased seizure susceptibility and other behavioral abnormalities of *Negr1*-KO mice. Apart from further neuroanatomical analyses, elucidating these issues requires the electrophysiological characterization of neuronal connectivity in *Negr1*-KO.

This accounts also for the neural circuits underlying impaired social behavior in *Negr1*-KO mice. The neuroanatomical substrates of social behavior comprises diverse subcortical structures reciprocally connected with the medial prefrontal cortex including the amygdala for emotional processing, the hypothalamus for stress modulation, the hippocampus for memory processing, the nucleus accumbens for social incentive, and regions of the cortex that process sensory and motor inputs and outputs (Ko, 2017). Previously, we and others have examined mice lacking the IgLON subgroup member LSAMP in the context of social behavior and spatial learning and memory (Qiu et al., 2010; Innos et al., 2011, 2013). Some of these findings are reminiscent to the results reported in the present study. The deficits in sociability and learning in both *Negr1*-KO and *Lsamp*-KO mice provide further evidence for the role of these genes in the genesis of abnormal behaviors. These mouse mutants may represent complementary models with value for studying the structural basis and the molecular mechanisms of behavioral abnormalities observed in many cognitive and psychiatric disorders.

## Author Contributions

KSingh, DL, BP, KH, KSchwald, HH, LM and MKES performed the experiments. JI, CJS, KR, OK, M-AP, CAH, KF, KL, BH, EV and MKES designed, supervised and analyzed the experiments. MKES led the project and wrote the manuscript supported by JI, CJS, CAH, KF, KL and BH. All authors have read and approved the final version of the manuscript.

## Conflict of Interest Statement

The authors declare that the research was conducted in the absence of any commercial or financial relationships that could be construed as a potential conflict of interest.
